# Association between insomnia and cognitive performance, gray matter volume, and white matter microstructure in cognitively unimpaired adults

**DOI:** 10.1186/s13195-019-0547-3

**Published:** 2020-01-07

**Authors:** Oriol Grau-Rivera, Grégory Operto, Carles Falcón, Gonzalo Sánchez-Benavides, Raffaele Cacciaglia, Anna Brugulat-Serrat, Nina Gramunt, Gemma Salvadó, Marc Suárez-Calvet, Carolina Minguillon, Álex Iranzo, Juan Domingo Gispert, José Luis Molinuevo, for the ALFA Study, Jordi Camí, Marta Crous-Bou, Carme Deulofeu, Ruth Dominguez, Xavi Gotsens, Laura Hernández, Gema Huesa, Jose M. González-de-Echávarri, Jordi Huguet, María León, Paula Marne, Eider M. Arenaza-Urquijo, Tania Menchón, Marta Milà, Maria Pascual, Albina Polo, Sandra Pradas, Aleix Sala-Vila, Sabrina Segundo, Mahnaz Shekari, Anna Soteras, Laia Tenas, Marc Vilanova, Natàlia Vilor-Tejedor

**Affiliations:** 1 Barcelonaβeta Brain Research Center (BBRC), Pasqual Maragall Foundation, Wellington 30, 08003 Barcelona, Spain; 2 Servei de Neurologia, Hospital del Mar, Barcelona, Spain; 3 Centro de Investigación Biomédica en Red de Bioingeniería, Biomateriales y Nanomedicina (CIBER-BBN), Madrid, Spain; 4 CIBER Fragilidad y Envejecimiento Saludable (CIBERFES), Madrid, Spain; 5 Neurology Service, Hospital Clínic de Barcelona and Institut D’Investigacions Biomèdiques August Pi i Sunyer, Barcelona, Spain; 6 Centro de Investigación Biomédica en Red sobre Enfermedades Neurodegenerativas (CIBERNED), Hospital Clínic de Barcelona, Barcelona, Spain; 7 Universitat Pompeu Fabra, Barcelona, Spain; 8 IMIM (Hospital del Mar Medical Research Institute), Barcelona, Spain

**Keywords:** Sleep, Insomnia, Neurocognitive disorders, Alzheimer disease, Inflammation, Neuropsychology, Magnetic resonance imaging, Voxel-based morphometry, Diffusion-weighted imaging

## Abstract

**Background:**

Mounting evidence links poor sleep quality with a higher risk of late-life dementia. However, the structural and cognitive correlates of insomnia are still not well understood. The study aims were to characterize the cognitive performance and brain structural pattern of cognitively unimpaired adults at increased risk for Alzheimer’s disease (AD) with insomnia.

**Methods:**

This cross-sectional study included 1683 cognitively unimpaired middle/late-middle-aged adults from the ALFA (ALzheimer and FAmilies) study who underwent neuropsychological assessment, T1-weighted structural imaging (*n* = 366), and diffusion-weighted imaging (*n* = 334). The World Health Organization’s World Mental Health Survey Initiative version of the Composite International Diagnostic Interview was used to define the presence or absence of insomnia. Multivariable regression models were used to evaluate differences in cognitive performance between individuals with and without insomnia, as well as potential interactions between insomnia and the *APOE* genotype. Voxel-based morphometry and tract-based spatial statistics were used to assess between-group differences and potential interactions between insomnia and the *APOE* genotype in gray matter volume and white matter diffusion metrics.

**Results:**

Insomnia was reported by 615 out of 1683 participants (36.5%), including 137 out of 366 (37.4%) with T1-weighted structural imaging available and 119 out of 334 (35.6%) with diffusion-weighted imaging. Individuals with insomnia (*n* = 615) performed worse in executive function tests than non-insomniacs and displayed lower gray matter volume in left orbitofrontal and right middle temporal cortex, bilateral precuneus, posterior cingulate cortex and thalamus, higher gray matter volume in the left caudate nucleus, and widespread reduction of mean and axial diffusivity in right hemisphere white matter tracts. Insomnia interacted with the *APOE* genotype, with *APOE*-ε4 carriers displaying lower gray matter volumes when insomnia was present, but higher volumes when insomnia was not present, in several gray matter regions, including the left angular gyrus, the bilateral superior frontal gyri, the thalami, and the right hippocampus.

**Conclusions:**

Insomnia in cognitively unimpaired adults at increased risk for AD is associated to poorer performance in some executive functions and volume changes in cortical and subcortical gray matter, including key areas involved in Alzheimer’s disease, as well as decreased white matter diffusivity.

## Introduction

Insomnia is a sleep-wake disorder characterized by difficulty initiating or maintaining sleep, along with an impairment of daytime functioning [1, 2] whose prevalence in the general population oscillates from 4 to 20%, according to different series [3–5].

Longitudinal epidemiological studies have linked poor sleep quality with a higher risk of late-life dementia [6, 7], and sleep fragmentation has been associated with a higher incidence of Alzheimer’s disease (AD) [8]. Understanding how insomnia and other causes of sleep disruption generate a higher vulnerability for AD constitutes a focus of major interest, given the potential of sleep quality as a therapeutic target for dementia prevention.

Increasing evidence suggests that sleep deprivation promotes the accumulation of β-amyloid and tau in the brain, which may be an important mechanism linking sleep disturbances and cognitive impairment [9, 10]. However, other mechanisms may drive this association. For instance, brain structural differences in individuals with poor sleep quality may contribute to lower the threshold for cognitive impairment [11–13]. In support of this hypothesis, previous neuroimaging studies have described lower gray matter volume involving well-known AD-vulnerable regions, such as precuneus, hippocampus, and cingulate gyrus in patients with insomnia [12, 14–18]. In addition, two independent studies have found, respectively, that poor sleep quality is associated to a higher rate of cortical atrophy [19] and reduced volume in brain regions usually affected in mild cognitive impairment and AD [13] in cognitively unimpaired adults. However, these studies have not evaluated potential interactions between sleep quality and *APOE* genotype, although previous evidence suggests that sleep quality interacts with *APOE* genotype in determining the risk of AD and the burden of β-amyloid and tau pathology in the brain [20, 21].

On the other hand, diffusion tensor imaging studies have shown decreased fractional anisotropy (which denotes microstructural integrity loss) in several white matter tracts in patients with insomnia and community-dwelling individuals with self-reported poor sleep quality [11, 22, 23].

In the present study, we aimed to characterize the pattern of cognitive performance, gray matter morphometry, and white matter microstructure associated with the presence of insomnia in a cohort of middle/late-middle-aged cognitively unimpaired individuals from the ALFA (ALzheimer and FAmilies) study [24]. Notably, the sample used in the present study has been enriched with AD risk factors, therefore potentiating possible associations between sleep quality and AD-related brain changes. We hypothesize that individuals with insomnia will display poorer performance in neuropsychological tests, lower brain volume involving areas usually involved in AD and altered white matter microstructure compared with non-insomniacs, with a more deleterious effect of insomnia being expected among *APOE*-ε4 carriers.

## Methods

### Participants

**Fig. 1:**
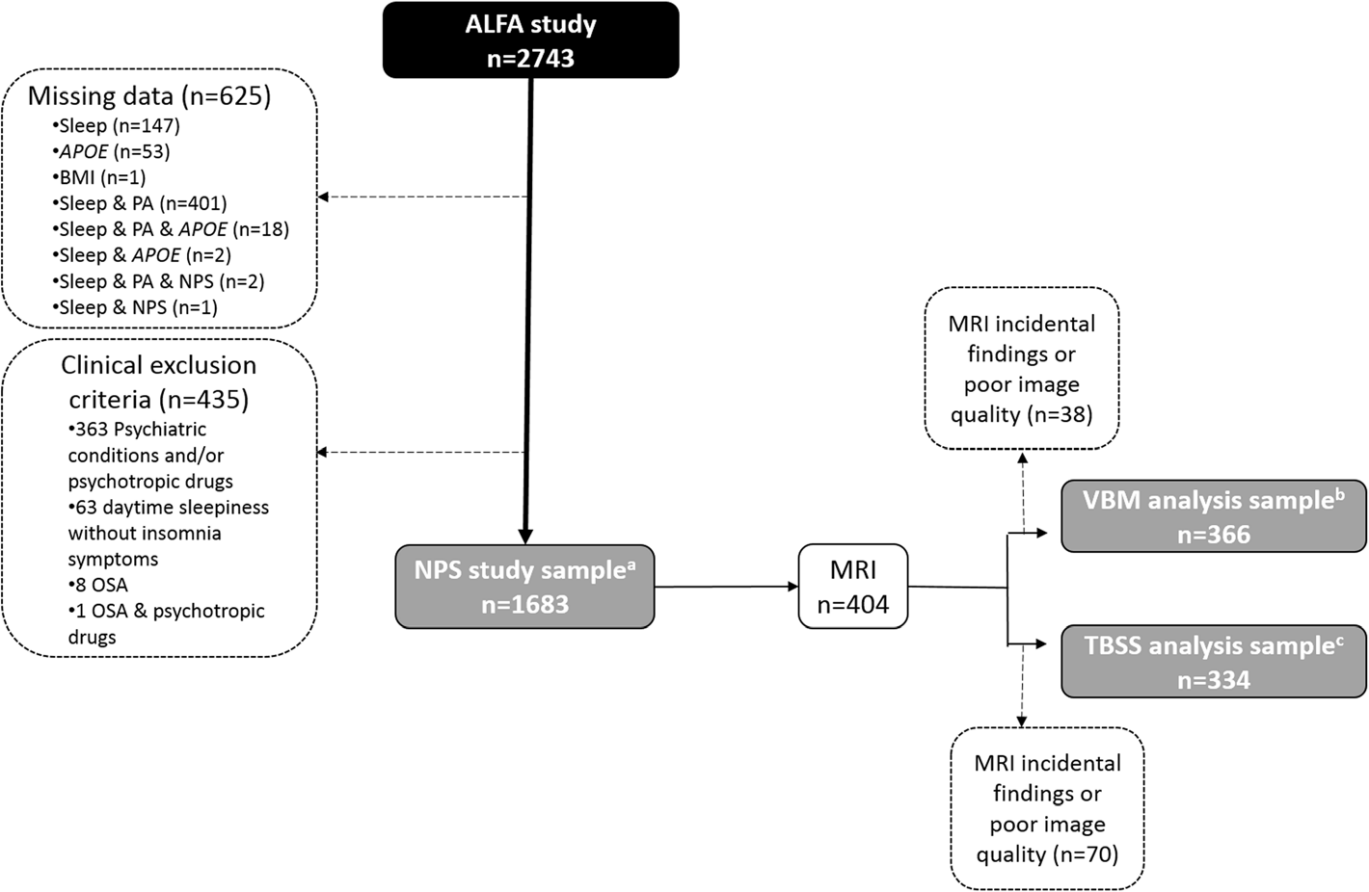
Participants’ selection flow-chart. ^a^This sample was used for analyses assessing associations between the presence of insomnia and performance in neuropsychological tests (NPS). ^b^This sample was used for analyses assessing associations between the presence of insomnia and gray matter volume. ^c^This sample was used for analyses assessing associations between the presence of insomnia and white matter diffusion imaging parameters

Participants were selected from the ALFA study cohort, which has been enriched for AD risk factors in terms of family history and *APOE*-ε4 genotype, and whose aim is to identify potential biomarkers and characterize early pathophysiological changes related to AD [24]. This study includes 2473 cognitively unimpaired adults (Clinical Dementia Rating = 0 and performance in the normal range in a screening neuropsychological test battery), mostly offspring of AD patients, aged between 45 and 75, recruited from the general population. The ALFA study excludes participants with current major depression or general anxiety disorder, bipolar disorder, schizophrenia, severe auditory and/or visual disorder, neurodevelopmental and/or psychomotor disorder, history of severe renal or hepatic insufficiency, chronic pneumopathy with long-term domiciliary oxygen, solid organ transplantation, fibromyalgia, active cancer, acquired cerebral damage, uncontrolled epileptic seizures, neurodegenerative disease, multiple sclerosis, or any other medical condition that might interfere in normal cognitive performance, as well as family history consistent with autosomal dominant AD. For the present study, we additionally excluded subjects with any psychiatric condition (besides those specified in the ALFA study exclusion criteria), current use of psychotropic medication, sleep-wake disorders other than insomnia, or missing data, rendering a study sample of 1683 participants (Fig. 1). Of these, 404 underwent magnetic resonance image (MRI) and had suitable images either for morphometric analyses (*n* = 366) and/or diffusion-weighted imaging (DWI) analyses (*n* = 334) (Fig. 1). The study was approved by the Ethics Committee of the “Parc de Salut Mar” (Barcelona, Spain).

### Sleep assessment

The presence of insomnia was assessed with the Spanish version of the World Mental Health Survey Initiative Version of the World Health Organization Composite International Diagnostic Interview (WMH-CIDI) [25, 26]. The CIDI is a standardized instrument intended for use in epidemiological studies that can generate diagnoses according to the DSM-IV and ICD-10 criteria. The following questions, included in the WMH-CIDI, were asked to all participants: “Did you have a period lasting two weeks or longer in the past 12 months when you experienced any of the following: (1) Problems getting to sleep, when nearly every night it took you two hours or longer before you could fall asleep (2) Problems staying asleep, when you woke up nearly every night and took an hour or more to get back to sleep (3) Problems waking up too early, when you woke up nearly every morning at least two hours earlier than you wanted to”. Insomnia was categorized as present if at least one of these questions was positively answered, or absent if all answers were negative. The self-reported number of sleep hours in a day was also registered for all participants. An additional question from the CIDI-WHM (“Did you have a period lasting two weeks or longer in the past 12 months when you experienced problems feeling sleepy during the day”) was used to screen for other potentially undiagnosed sleep-wake disorders in the control group (e.g., obstructive sleep apnea), and those participants without insomnia who answered positively to this question were excluded from the study (Fig. 1).

### Neuropsychological and mood evaluation

The Spanish version of the Memory Binding Test (MBT) [27] was used to evaluate episodic memory. The MBT includes four variables: immediate total paired recall (TPR), immediate total free recall (TFR), delayed total paired recall (TDPR), and delayed total free recall (TDFR), which evaluate free and cued recalls in immediate and delayed trials. Executive functions were assessed with five WAIS-IV subtests [28]: Digit Span (measure of immediate and working memory); Coding subtest (measure of processing speed and attention); Matrix Reasoning and Visual Puzzles (measures of fluid intelligence, logic and executive functioning, and visual reasoning, respectively); and Similarities (measure of abstract verbal reasoning). Anxiety and depressive symptoms were measured by summing the scores of each component of the Goldberg Anxiety and Depression Scale (GADS) [29].

### Image acquisition and processing

MRI scans were performed using a 3-T General Electric Discovery scanner. High-resolution 3D structural T1-weighted images were obtained using a fast spoiled gradient-echo sequence with the following parameters: Repetition time = 6.16 ms, echo time = 2.33 ms, inversion time = 450 ms, flip angle 12°, matrix size = 256 × 256 × 174, and voxel size = 1 mm^3^ isotropic. The DWI protocol consisted in an echo-planar imaging sequence with 64 diffusion-encoding directions (*b* = 1000 s mm ^−2^) and one T2-weighted baseline (*b* = 0), a field of view of 256 × 256 mm, and an imaging matrix of 128 × 128 with 56 slices (thickness = 2 mm) and 2-mm isotropic voxels. Gray matter segmentation was performed with SPM12 (Statistical Parametric Mapping, Welcome Trust Centre for Neuroimaging, UK; http://www.fil.ion.ucl.ac.uk/spm). T1 images were normalized to the Montreal Neurological Institute space using DARTEL and smoothed with a 6-mm full-width at half maximum Gaussian kernel [30]. DWI images were denoised and corrected for eddy current distortion as described elsewhere [31], then analyzed using FMRIB Software Library (FSL; http://www.fmrib.ox.ac.uk/fsl). Fractional anisotropy (FA), mean (MD), axial (AxD), and radial diffusivity (RD) maps were obtained with DTIFit. Skeletonized maps were generated with Tract-Based Spatial Statistics (TBSS) [32]. A group mean FA image was used to generate the mean FA skeleton (binarized with FA > 0.2 threshold). Aligned FA, MD, AxD, and RD data from each subject were projected onto this skeleton. Voxel-wise general linear model statistics were fed with the resulting data. The same workflow was applied to each parametric map. Significant clusters were anatomically labeled using the Johns Hopkins University tract-based white matter atlas [33].

### Statistical analyses

Differences in demographic and clinical variables between individuals with and without insomnia, as well as differences in the prevalence of insomnia based on the *APOE* status (ε4 non-carrier, ε4 heterozygous, or ε4 homozygous), were evaluated with two-sided *t* test or chi-squared test. We also performed a logistic regression to calculate the odds of having insomnia as a function of the *APOE* status while adjusting by age and sex. Potential confounders for those analyses evaluating the effect of insomnia in different outcomes were selected a priori based on well-known risk factors for sleep disturbances and/or cognitive impairment, as well as other variables associated to brain function and/or structure, such as cardiovascular risk factors [34–36], *APOE ε4* allele carriership [37–40], body mass index (BMI) [41–43], and the level of anxiety and depression [36, 44, 45], among others. Associations between the presence of insomnia and cognitive performance were first evaluated with a multivariable linear regression model with a *p* value threshold for statistical significance of *p* < 0.005 using a Bonferroni-type correction (≈ 0.05 divided by nine cognitive outcomes). Gray matter volume between-group differences were evaluated with two-sample *t* test using voxel-based morphometry (VBM) and the general linear model method implemented in SPM12. As we expected a small effect size [14, 15], we used a liberal threshold of *p* < 0.005 uncorrected for multiple comparisons and a cluster-extent threshold (*k*) of 100 voxels and subsequently assessed whether any significant cluster survived family-wise error (FWE) correction for multiple comparisons (*p* < 0.05). For the TBSS analysis, the number of permutations was set at 5000. Statistical significance was set at *p* < 0.05, after FWE correction, using the threshold-free cluster enhancement option, implemented in FSL [46]. TBSS results were processed using tbss_fill script to aid visualization. All statistical analyses were adjusted by age, sex, education, number of *APOE-ε4* alleles, GADS, and BMI. VBM analyses were also adjusted by squared age (to account for non-linear effects) [38] and total intracranial volume. We did not adjust TBSS analyses for age squared, as we did not found evidence of a non-linear association between age and TBSS metrics in a previous work based on the same dataset [39]. Neither we included a quadratic term for age in cognitive performance analyses, as doing so did not substantially modify the models. We did not adjust the analyses for the presence of diabetes mellitus due to its low prevalence in our sample (< 4%) and balanced distribution among subjects with and without insomnia (Table 1).

**Table 1:** Demographic, genetic, and clinical characteristics in the entire sample

	Participant group			
Characteristics	All (*n* = 1683)	Controls (*n* = 1068)	Insomnia (*n* = 615)	*p*
Age, mean (SD), years	55.8 (6.7)	55.4 (6.5)	56.6 (6.8)	< .001
Female, no. (%)	1018 (60.5)	593 (55.5)	425 (69.1)	< .001
Education, mean (SD), years	13.6 (3.5)	13.8 (3.5)	13.2 (3.5)	.002
APOε4 allele status, no. (%)				
Non-carriers	1108 (65.8)	703 (65.8)	405 (65.9)	
Heterozygotes	515 (30.6)	321 (30.1)	194 (31.5)	.246
Homozygotes	60 (3.6)	44 (4.1)	16 (2.6)	
BMI, mean (SD), kg/m^2^	26.6 (4.2)	26.5 (4.1)	26.7 (4.3)	.406
Sleep duration, hours (SD)	7 (0.8)	7.2 (0.8)	6.7 (0.9)	< .001
GADS (SD)	0.6 (1.4)	0.5 (1.1)	1.0 (1.7)	< .001
Physically active, no. (%)	1057 (62.8)	674 (63.1)	383 (62.3)	.734
Hypertension, no. (%)	308 (18.3)	191 (17.9)	117 (19.0)	.560
Dyslipidemia, no. (%)	497 (29.5)	300 (28.1)	197 (32.0)	.088
Diabetes mellitus, no. (%)	59 (3.5)	40 (3.8)	19 (3.1)	.481

*BMI* body mass index, *GADS* Sum of Goldberg Anxiety and Depression Scale scores

We also evaluated potential interactions between insomnia and *APOE* status, in cognitive performance, white matter diffusivity, and gray matter volume. For analyses evaluating the effect of this interaction on cognitive performance, we built different models assuming different potential genetic effects. Thus, *APOE* status (ε4 non-carriers/ε4 heterozygous/ε4 homozygous) was included in regression models as a continuous variable and coded as 0/1/2 in the additive model, as 0/1/1 in the dominant model, and as 0/0/1 in the recessive model. Significance threshold for these analyses was set at *p* < 0.0019 (≈ 0.05 divided by nine cognitive outcomes × three genetic models). For neuroimaging analyses, we included six dummy regressors accounting for all possible combinations between *APOE* status and the presence or absence of insomnia, and separate *t* tests contrast weights were specified to account for the different genetic models.

### Supplementary analyses

In order to exclude other potential sources of bias, we performed a supplementary analysis controlling by additional confounders [self-reported hypertension, dyslipidemia, and level of physical activity (defined as “active” if engaged in moderate physical activity at least 150 min/week or vigorous physical activity at least 75 min/week, or “inactive” otherwise)].

## Results

Six hundred fifteen participants fulfilled the criteria used for insomnia (36.5%). Compared with non-insomniacs (controls), these participants were older, reported less years of education and shorter sleep duration, scored higher in GADS, and included a higher percentage of women (Table 1).

In turn, insomnia was more frequent in women (41.8%) than men (28.6%). Among individuals with insomnia, 25.3% reported only difficulties maintaining sleep, 19.4% reported only early morning awakening, 9.6% reported only difficulties initiating sleep, and 45.7% reported more than one insomnia symptom (21.5% difficulties maintaining sleep and early morning awakening, 15% difficulties initiating and maintaining sleep and early morning awakening, and 9.2% difficulties initiating and maintaining sleep). *APOE*-ε4 homozygotes had a lower prevalence of insomnia (26.7%), compared with heterozygotes (37.7%) and non-carriers (36.6%), although this difference was not statistically significant and could be explained by the homozygous younger mean age. An age- and sex-adjusted logistic regression did not show any significant association between *APOE*-ε4 status and the odds of having insomnia. These characteristics were very similar in the subsample with MRI, except for an overall higher percentage of *APOE-ε4* allele carriers (which was balanced among individuals with and without insomnia) due to the ALFA study recruitment strategy (Table 2).

**Table 2:** Demographic, genetic, and clinical data in the MRI subsample

	Gray matter volume (VBM) analyses (*n* = 366)			White matter DWI (TBSS) analyses (*n* = 334)		
	Controls	Insomnia	*p*	Controls	Insomnia	*p*
Sample size, no. (%)	229	137	–	215	119	–
Age, years (SD)	56.7 (7.4)	56.7 (7.0)	.917	56.8 (7.6)	56.8 (6.9)	.962
Female, no. (%)	117 (51.1)	87 (63.5)	.021	111 (51.6)	76 (63.6)	.036
Education, years (SD)	13.9 (3.6)	13.7 (3.6)	.567	13.9 (3.6)	13.7 (3.6)	.648
*APOE*-ε4 allele status, no. (%)						
Non-carriers	118 (51.5)	69 (50.4)	.391	108 (50.2)	62 (51.7)	.108
Heterozygotes	78 (34.1)	54 (39.4)		74 (34.4)	48 (40.7)	
Homozygotes	33 (14.4)	14 (10.2)		33 (15.4)	10 (7.6)	
BMI, mean (SD), kg/m2	26.9 (4.3)	26.5 (3.8)	.353	26.8 (4.3)	26.5 (3.8)	.639
GADS (SD)	0.4 (0.98)	0.96 (1.7)	< .001	0.41 (1.0)	1.0 (1.7)	< .001
Hypertension, no. (%)	51 (22.3)	27 (19.7)	.562	44 (20.5)	23 (19.5)	.832
Dyslipidemia, no. (%)	64 (28.0)	42 (30.1)	.580	58 (27.0)	38 (32.2)	.314
Diabetes mellitus, no. (%)	7 (3.1)	3 (2.2)	.622	6 (2.8)	3 (2.5)	0.894
Physically active, no. (%)	141 (61.6)	95 (69.3)	.133	130 (60.5)	82 (69.5)	.101
Sleep duration, hours (SD)	7.2 (0.70)	6.7 (0.93)	< .001	7.2 (0.7)	6.7 (0.9)	< .001
Total intracranial volume (SD)	1520.4 (139.5)	1471.3 (156.7)	.002	1517.8 (136.5)	1474.0 (158.2)	0.009

*BMI* body mass index, *GADS* Sum of Goldberg Anxiety and Depression Scale scores

### Cognitive performance analyses

Multivariable linear regression analyses showed a significant negative association between the presence of insomnia and performance in WAIS-IV Digit Span. A negative association was also observed with WAIS-IV Coding, but this association did not survive multiple comparisons correction (Table 3). Results did not significantly change after including hypertension, dyslipidemia, and physical activity as additional covariates in the model (Table 3).

**Table 3:** Effect of insomnia on cognitive performance

	*β* coefficient (95% confidence interval)	*p*	Adjusted *R*^2^
Model 1			
MBT-TFR	− 0.24 (− 0.72, 0.23)	.316	0.122
MBT-TPR	0.04 (− 0.38, 0.46)	.850	0.085
MBT-TDFR	− 0.24 (− 0.72, 0.25)	.332	0.131
MBT-TDPR	− 0.08 (− 0.52, 0.35)	.713	0.096
WAIS-IV Coding	− 1.28 (− 2.54, − 0.02)	.046	0.281
WAIS-IV Visual Puzzles	− 0.21 (− 0.61, 0.18)	.290	0.201
WAIS-IV Digit Span	− 0.75 (− 1.26, − 0.24)	.004	0.140
WAIS-IV Matrix Reasoning	− 0.17 (− 0.55, 0.22)	.398	0.234
WAIS-IV Similarities	0.31 (− 0.11, 0.73)	.145	0.220
Model 2			
MBT-TFR	− 0.24 (− 0.71, 0.23)	.320	0.121
MBT-TPR	− 0.04 (− 0.38, 0.46)	.852	0.084
MBT-TDFR	− 0.24 (− 0.72, 0.25)	.337	0.131
MBT-TDPR	− 0.08 (− 0.52, 0.35)	.710	0.096
WAIS-IV Coding	− 1.28 (− 2.54, − 0.02)	.046	0.281
WAIS-IV Visual Puzzles	− 0.21 (− 0.60, 0.18)	.297	0.201
WAIS-IV Digit Span	− 0.75 (− 1.25, − 0.24)	.004	0.139
WAIS-IV Matrix Reasoning	− 0.17 (− 0.55, 0.22)	.395	0.233
WAIS-IV Similarities	0.31 (− 0.11, 0.73)	.144	0.223

Model 1: adjusted by age, sex, education, number of *APOE-ε4* alleles, GADS, and BMI. Model 2: same covariates than model 1 and additionally adjusted by self-reported hypertension, dyslipidemia, and level of physical activity

We found a trend for interactions between *APOE*-ε4 status and insomnia in WAIS-IV Matrix Reasoning test (*p* = 0.042 in the additive model), in WAIS-IV Digit Span test (*p* = 0.036 in the dominant model), and in MBT-TDFR (*p* = 0.034 in the recessive model), but none of them survived correction for multiple comparisons. When performing stratified analyses by *APOE*-ε4 to test the association between insomnia and cognitive performance in those domains where a trend for an interaction was found, we found a detrimental effect of insomnia on MBT-TDFR performance (*p* = 0.036) exclusively in *APOE*-ε4 homozygous, and a detrimental effect of insomnia only in *APOE*-ε4 non-carriers on WAIS-IV Digit Span performance (*p* < 0.001) (Additional file 1: Figure S1). No significant associations between insomnia and WAIS-IV Matrix Reasoning test performance showed up in any of the *APOE* groups.

### VBM analysis

**Fig. 2:**
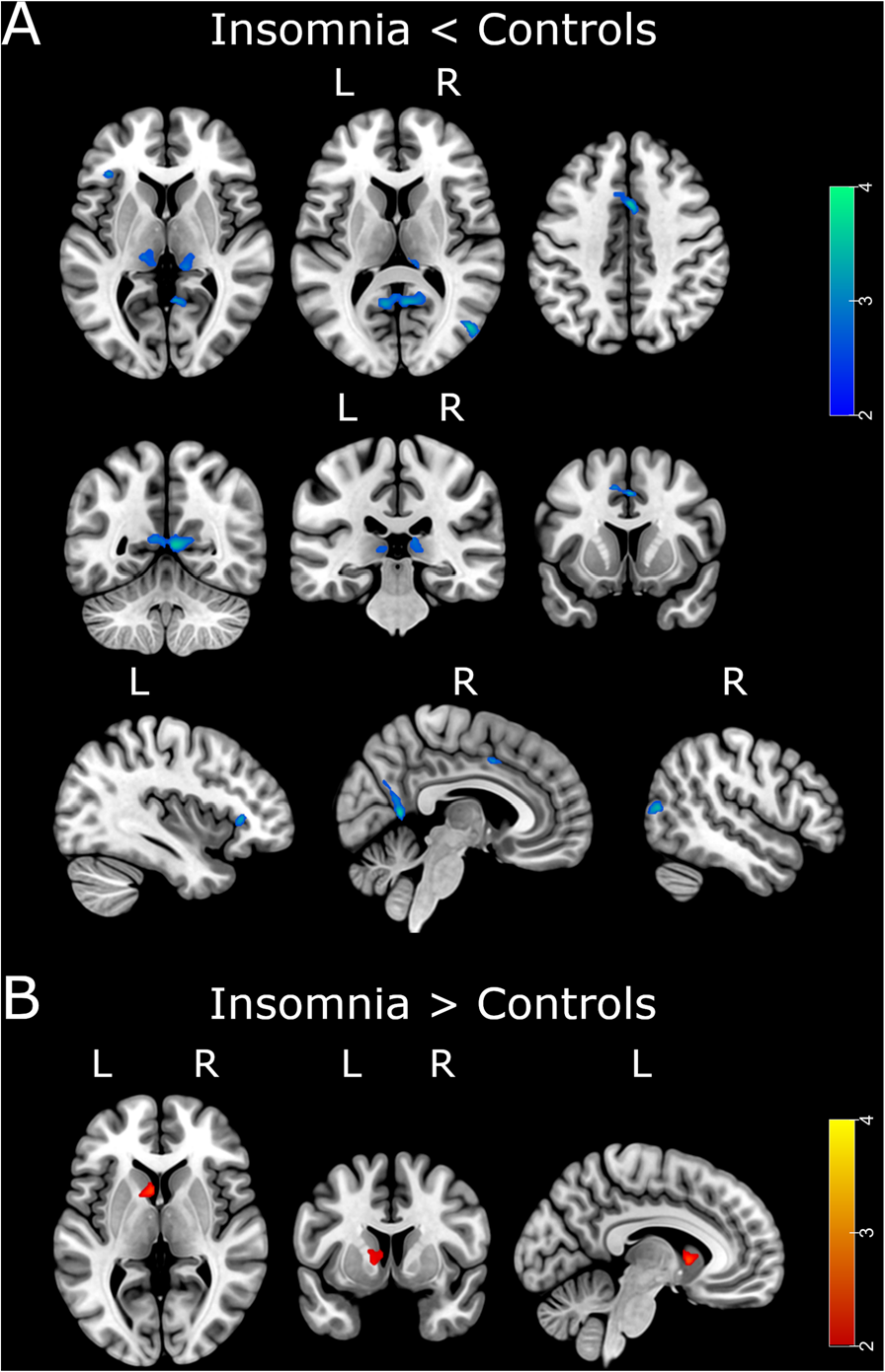
Effect of insomnia on gray matter volume. **a** Blue-green colored regions show areas with significantly lower volume in participants with insomnia compared with controls (*p*_uncorrected_< 0.005; *k* = 100). **b** Red-yellow colored areas show areas with significantly higher brain volume in participants with insomnia compared with controls. L, left hemisphere; R, right hemisphere

Participants with insomnia displayed significantly lower volume in the left orbitofrontal cortex, bilateral posterior cingulate cortex, bilateral precuneus, bilateral middle cingulum, right middle temporal gyrus, and bilateral thalamus, as well as higher volume in the left caudate nucleus (Fig. 2, Table 4).

**Table 4:** VBM results of the main effect of insomnia (*p*_uncorrected_ < 0.005; *k* = 100)

		Cluster size (*k*)	Peak-level *t* value		MNI coordinates		
Contrast	Anatomical area			*P*_uncorrected_	*x*	*y*	*z*
I < C	Bilateral PCC/precuneus	557	3.78	< .001	6	− 56	7.5
	Bilateral middle cingulum	176	3.64	< .001	2	9	42
	Right middle temporal	156	3.60	< .001	48	− 72	11
	Right thalamus	154	2.97	.002	12	− 33	5
	Left thalamus	114	2.97	.002	− 15	− 26	3
	Left orbitofrontal	103	3.43	< .001	− 41	29	3
I > C	Left caudate	181	3.02	.001	− 6	9	3

*I* insomnia, *C* controls, *PCC* posterior cingulate cortex

None of these clusters survived FWE multiple comparison correction. Results were similar after adding hypertension, dyslipidemia, and physical activity as additional covariates to the model (Additional file 1: Figure S2, Table S1). However, we observed an overall modest size decrease in most significant clusters, with some of them not surviving the *k* = 100 threshold [left thalamus (*k* = 95) and left orbitofrontal cortex (*k* = 88)].

**Fig. 3:**
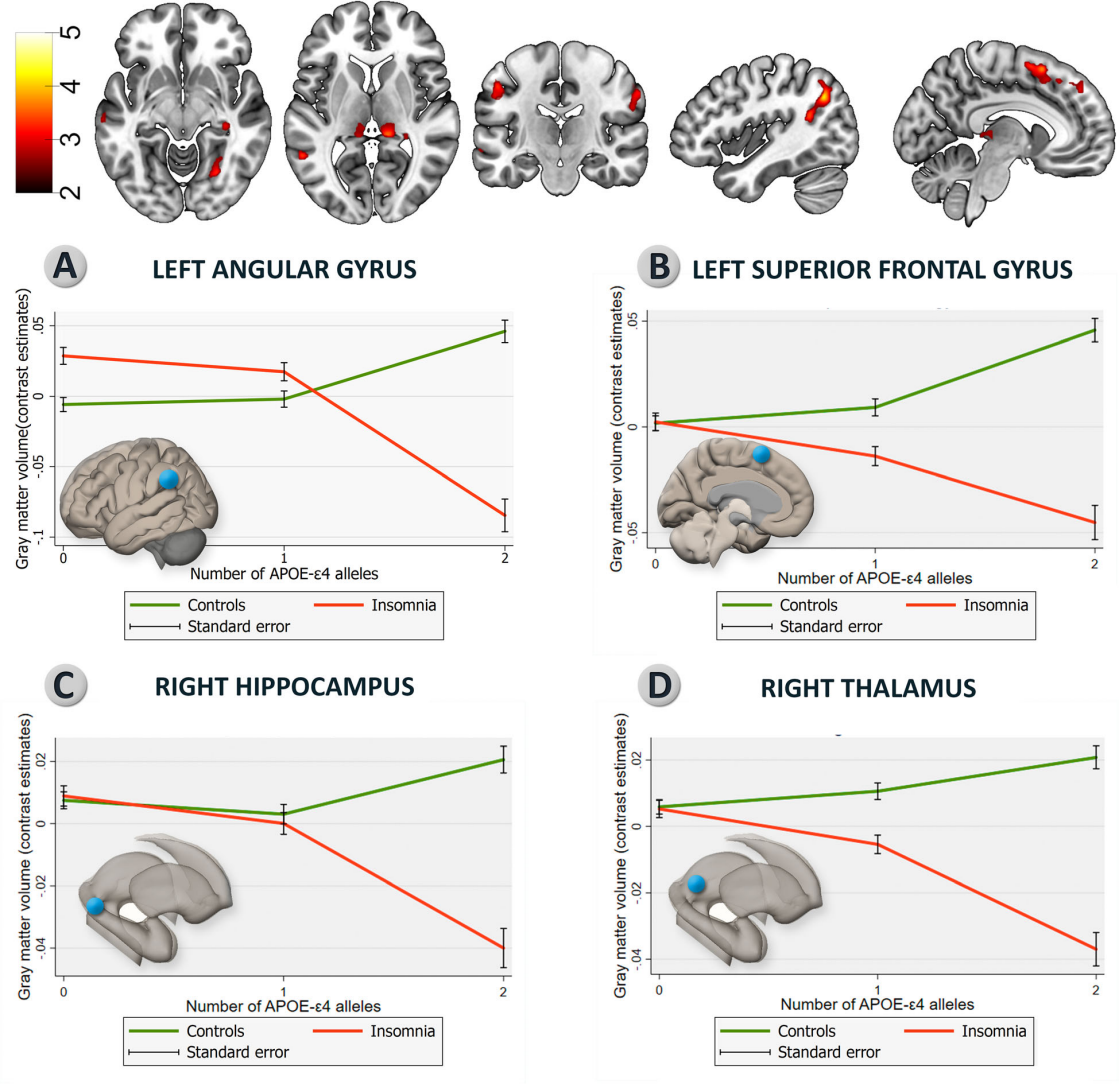
Interactions between APOE-ε4 status and insomnia in gray matter volume. **a** Gray matter areas where a significant interaction between APOE-ε4 status and insomnia was found (puncorrected < 0.005; k = 100, only the additive model is shown). Graphs **b**–**e** show how the association between APOE status and gray matter volume is modulated by the presence of insomnia in four representative brain regions. L, left hemisphere; R, right hemisphere

VBM analyses returned a significant interaction between insomnia and *APOE*-ε4 status in several gray matter regions including the left angular, left middle temporal, bilateral superior frontal, left fusiform, and bilateral postcentral gyri, as well as the thalami and the right hippocampus (Fig. 3, Additional file 1: Table S2). In these regions, divergent structural patterns were observed among individuals with different *APOE*-ε4 status depending on the presence or absence of insomnia (Fig. 3). Thus, homozygous showed lower or higher gray matter volumes depending on the presence or absence of insomnia, respectively. A similar divergent structural pattern was observed in some significant clusters among heterozygous individuals, but the effect size was milder and it was not consistently observed in all brain regions. On the other hand, insomniac and non-insomnia non-carriers tended to show similar gray matter volumes in those regions where significant interactions were observed (Fig. 3).

### TBSS analysis

**Fig. 4:**
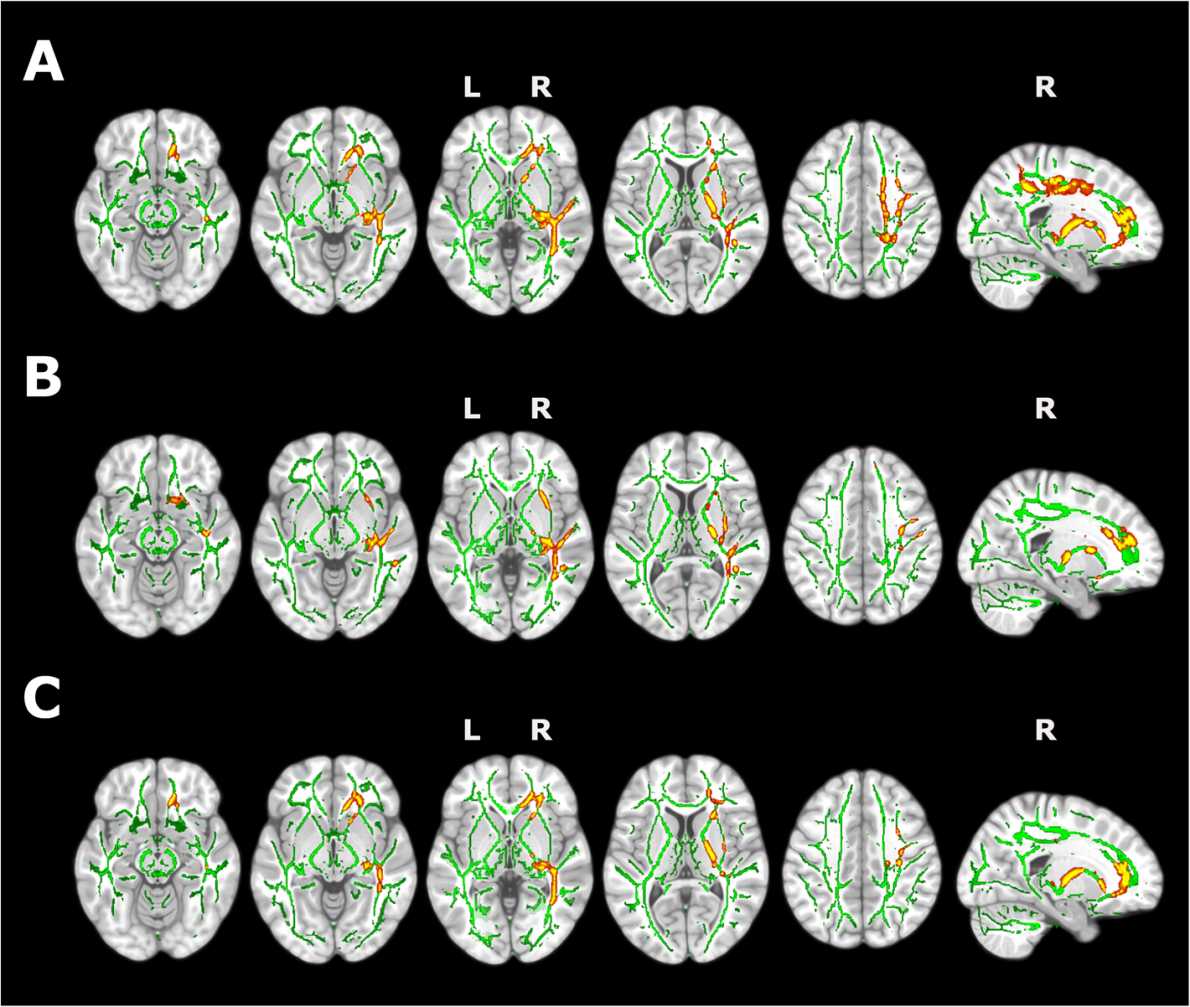
Effect of insomnia on white matter microstructure. Significant white matter clusters derived from tract-based spatial statistics are represented in red-yellow over the skeletonized white matter tracts (green). Individuals with insomnia showed significantly reduced values of mean (**a**) and axial (**b**) diffusivity (FWE corrected p value < 0.05), and a trend for radial diffusivity (**c**) (FWE corrected *p* value between 0.05 and 0.1), compared with normal sleepers. L, left hemisphere; R, right hemisphere

Insomnia was associated to significantly lower MD values in white matter from the right hemisphere, involving tracts from corona radiata (anterior, superior, and posterior), internal capsule (anterior and posterior limbs and retrolenticular part), external capsule, superior and inferior longitudinal fasciculus, superior and inferior fronto-occipital fasciculus, corpus callosum, posterior thalamic radiation, fornix/stria terminalis, and cerebral peduncle (Fig. 4). In most of these regions, AxD was also reduced in subjects with insomnia. We also found a trend for reduced RD (FWE corrected *p* value between 0.05 and 0.1) overlapping with those WM tracts with decreased MD and AxD. We did not find any significant increase in MD, AxD, or RD in participants with insomnia, or any significant between-group difference in FA values. Additional adjustment for hypertension, dyslipidemia, and physical activity did not modify these results (Additional file 1: Figure S3).

We did not find any significant interaction between *APOE*-ɛ4 status and insomnia in white matter diffusion metrics.

## Discussion

In the present study involving cognitively unimpaired individuals at increased risk for AD, we found that insomnia was associated to poorer performance in some executive functions and to a distinctive brain macro- and microstructural pattern, characterized by cortical and subcortical GMv differences and decreased white matter diffusivity. In addition, we found that the association between insomnia and gray matter volume is modulated by the *APOE*-ε4 status, so that *APOE*-ε4 carriers tend to display lower gray matter volumes in the presence of insomnia, but higher volumes when insomnia is not present.

The demographic and clinical profile of participants with insomnia from this study is similar to that reported in individuals with poor sleep quality from population-based studies [47, 48]. However, by using a study sample enriched for AD risk factors, we have been able to detect neuroimaging findings that differ from those reported in previous studies, particularly those showing decreased mean and axial diffusivity in white matter tracts, which may be relevant for understanding the association between poor sleep quality and AD in individuals at higher risk for this disease.

Several studies have analyzed the cognitive correlate of insomnia or poor sleep quality, leading in some cases to inconsistent results [47–50]. Our findings are in line with a meta-analysis of 24 studies showing worse performance in executive functions among individuals with insomnia [50]. Subsequent studies have also reported altered executive functions in patients with insomnia [51, 52] and community-dwelling individuals with self-reported poor sleep quality [53]. We did not find differences in episodic memory performance, despite previous evidence of poorer memory performance among individuals with insomnia [50]. This could be explained by a selection bias towards patients with more severe insomnia (possibly linked to worse cognitive performance) in studies conducted at Sleep Units. Also, we cannot exclude an effect of insomnia on other cognitive domains, such as language and visuoperceptual or visuospatial abilities, as they were not evaluated in our study. Regarding the relationship between cognitive and neuroimaging findings, our group previously described a positive correlation between speed processing and thalamic, as well as superior longitudinal fasciculus (SLF) volume in cognitively unimpaired adults, which is consistent with our results showing a trend towards lower processing speed, as well as lower thalamic volume and altered diffusivity in the SLF in insomniacs [54].

Regarding the interaction analyses in cognitive performance, although our findings did not survive correction for multiple comparisons, the potential interaction between *APOE* and insomnia in delayed episodic memory performance deserves further study, as it is consistent with our finding of lower hippocampal volume in *APOE*-ɛ4 carriers with insomnia (as opposed to non-insomniac *APOE*-ɛ4 carriers), considering the pivotal role of the hippocampus in episodic memory formation [55], and it is also in line with previous evidence of a negative interaction between *APOE*-ɛ4 and sleep disturbance on memory performance [56].

Our findings of lower gray matter volume in orbitofrontal and parietal cortex, as well as middle cingulate gyrus, recapitulate some of the main brain volume differences previously reported in patients with insomnia [14, 15, 17, 18, 57, 58], which supports the existence of a brain structural signature associated to this condition. Regarding possible mechanistic links between these alterations and poor sleep quality, it has been hypothesized that orbitofrontal cortex abnormalities may predispose to insomnia due to altered sensing of the optimal temperature for sleep [14, 59, 60]. As far as we are concerned, lower thalamic volume has not been previously reported in patients with insomnia, although it has been associated with increased sleep fragmentation variability in cognitively unimpaired elderly subjects [61]. Thalamic involvement in sleep disturbances is biologically plausible, since regulation of wakefulness and sleep cycles largely relies on a neural network involving neurons in the brainstem, hypothalamus and basal forebrain that provides excitatory input to the thalami and cortical regions [62]. Also, degeneration of this nucleus in familial and sporadic fatal insomnia, a rare subtype of prion diseases, leads to prominent sleep disturbances [63].

Our finding of lower gray matter volume in precuneus and posterior cingulate cortex in individuals with insomnia could be linked to the higher vulnerability for cognitive impairment that has been observed in association with poor sleep quality, as these regions are early involved in AD [64]. Considering that poor sleep quality has been associated with higher levels of β-amyloid deposition in the brain [65–67], changes in these regions (i.e., precuneus and posterior cingulate cortex) could be related to a higher prevalence of individuals with preclinical AD in the insomnia group. An alternative hypothesis is that structural differences observed in individuals with insomnia may represent pre-existing morphological traits that could confer a higher vulnerability to both insomnia and cognitive impairment.

Unexpectedly, we found a greater volume in the left caudate in individuals with insomnia. The interpretation of this finding should be considered with caution, as higher caudate volume has not been previously reported in individuals with insomnia. Even so, with these limitations in mind, one potential explanation would be the existence of a higher prevalence of individuals with preclinical AD among those with insomnia, based on previous evidence of increased caudate size in presymptomatic *PSEN1* mutation carriers, which is a genetic cause of AD [68], and previous findings suggest a transient size increase in some brain structures during early stages of AD [69, 70].

Our interaction analyses point to *APOE* as a potential modulator in the association between sleep and brain structure, with a higher detrimental effect of insomnia on brain structure being observed among *APOE*-ɛ4 carriers. This is in line with previous evidence suggesting that *APOE*-ɛ4 carriers may be more vulnerable to different environmental factors, such as lifestyle and vascular risk factors [71], and also with a previous study showing that better sleep quality attenuates the effect of *APOE*-ɛ4 on AD incidence and neurofibrillary tangle burden [20].

On the other hand, we also found that *APOE*-ɛ4 carriers without insomnia tend to display higher gray matter volumes compared with non-carriers, which was not expected. Assuming the hypothesis that sleep quality is gradually deteriorated as AD pathology accumulates in the brain [72], a potential explanation for this finding would be that the presence of insomnia among *APOE*-ɛ4 carriers (which are more likely to harbor AD neuropathological change than non-carriers) may be associated with a more advanced stage in the preclinical phase, whereas *APOE*-ɛ4 carriers without insomnia may include a higher proportion of individuals in an earlier AD preclinical stage, where neuroinflammation may still overcome neurodegeneration [70], resulting in overall greater gray matter volumes in this group.

We found lower diffusivity values involving widespread white matter tracts, exclusively in the right hemisphere. Previous studies have shown a right predominant loss of white matter integrity in patients with insomnia [11, 22]. Studies in human healthy volunteers have reported an asymmetry in brain hemisphere activity during wakefulness (with left-hemisphere predominance) that is reversed during sleep [73, 74]. Whether differences in the laterality pattern across the sleep-wake cycle may be related to a higher vulnerability of the right hemisphere white matter tracts to insomnia-related disruption deserves further investigation. On the other hand, a critical difference between our findings and those reported in previous studies is that we found decreased, rather than increased diffusivity associated with insomnia [11, 23]. Acute ischemic lesions, tumoral lesions, and inflammation are among the main established causes of MD reduction in the brain tissue [75]. These three scenarios have in common a reduction in water molecules diffusivity due to their confinement to the intracellular compartment, either due to cellular swelling or cellular proliferation. Thus, one potential explanation for our findings is the existence of insomnia-related neuroinflammation involving white matter. In support of this hypothesis, a recent meta-analysis reported an association between insomnia and elevated systemic inflammatory markers [76], and murine model studies have shown that circadian clock disruption induces astrogliosis [77] and sleep disturbance is associated with higher expression of pro-inflammatory interleukins and microglial activation in mouse brains [78, 79]. An alternative explanation is that our results could have been driven by a hypothetical higher prevalence of preclinical AD among individuals with insomnia, as decreased MD in white matter has been previously associated with early β-amyloid deposition [80]. This could also explain the difference between our findings and those from previous studies, as our sample has been enriched for AD risk factors, therefore facilitating the detection of AD-related changes.

The main strengths of our study are the large size and characteristics of the study sample and its multimodal approach. Although using a sample enriched for AD risk factors may preclude the generalizability of our results, it is better suited to detect brain structural differences that might be driven by AD pathology. On the other hand, an important limitation of the present study is that we have used a subjective measure that queries for the core criteria of insomnia but does not provide more detailed information on sleep quality. In this sense, having used more specific subjective or objective sleep measures may have resulted in more robust statistical associations between sleep quality and neuroimaging and cognitive outcomes. The fact that gray matter volume differences did not survive correction for multiple comparisons (which is inherent to the small effect size of poor sleep quality on gray matter volume) and the lack of AD biomarkers are other relevant limitations in order to interpret our findings. However, we plan to address some of these questions in a further study including data from a longitudinal cohort nested in the ALFA study that incorporates CSF and PET biomarkers.

## Conclusions

In summary, we confirmed that insomnia is associated to a distinctive cognitive and brain structural pattern in cognitively unimpaired individuals at risk for AD. Importantly, our findings in white matter microstructure suggest that some brain structural differences associated to poor sleep quality may be mediated by neuroinflammation. Further studies should explore whether insomnia-related brain structural differences correspond to pre-existing traits conferring higher vulnerability for either insomnia and neurodegenerative diseases, or are related to primary sleep-deprivation effects and/or AD-related effects triggered by sleep disturbances.

## Supplementary information

**Additional file 1: Figure S1.** Interaction between APOE status and insomnia in cognitive performance. **Figure S2.** Effect of insomnia on gray matter volume (additionally adjusted by hypertension, dyslipidemia and physical activity). **Figure S3.** Effect of insomnia on white matter microstructure (additionally adjusted by hypertension, dyslipidemia and physical activity). **Table S1.** VBM results of the main effect of insomnia, additionally adjusted by hypertension, dyslipidemia and physical activity (puncorrected<0.005; k=100). **Table S2.** VBM results of interaction between insomnia and *APOE* genotype (puncorrected<0.005; k=100).

## Data Availability

The datasets used and/or analyzed during the current study are available from the corresponding author on reasonable request.

## Abbreviations

AD: Alzheimer’s disease
ALFA: ALzheimer and Families
AxD: Axial diffusivity
BMI: Body mass index
DWI: Diffusion-weighted imaging
FA: Fractional anisotropy
GADS: Goldberg Anxiety and Depression Scale
MBT: Memory Binding Test
MD: Mean diffusivity
MRI: Magnetic resonance imaging
NPS: Neuropsychological tests
OSA: Obstructive sleep apnea
PA: Physical activity
RD: Radial diffusivity
TBSS: Tract-based spatial statistics
TDFR: Delayed total free recall
TDPR: Delayed total paired recall
TFR: Immediate total free recall
TPR: Immediate total paired recall
VBM: Voxel-based morphometry
WMH-CIDI: World Health Organization Composite International Diagnostic Interview
