# Supplementary material for: Association between insomnia and cognitive performance, gray matter volume, and white matter microstructure in cognitively unimpaired adults
Source: Alzheimers Res Ther. 2020 Jan 7;12:4. doi: 10.1186/s13195-019-0547-3 (PMC6945611; doi:10.1186/s13195-019-0547-3)
Supplement: Supplementary file 1 — Additional file 1: Figure S1. Interaction between APOE status and insomnia in cognitive performance. Figure S2. Effect of insomnia on gray matter volume (additionally adjusted by hypertension, dyslipidemia and physical activity). Figure S3. Effect of insomnia on white matter microstructure (additionally adjusted by hypertension, dyslipidemia and physical activity). Table S1. VBM results of the main effect of insomnia, additionally adjusted by hypertension, dyslipidemia and physical activity (puncorrected<0.005; k=100). Table S2. VBM results of interaction between insomnia and APOE genotype (puncorrected<0.005; k=100). [file 13195_2019_547_MOESM1_ESM.pdf]

Additional file 1

Figure S1. Interaction between APOE status and insomnia in cognitive performance

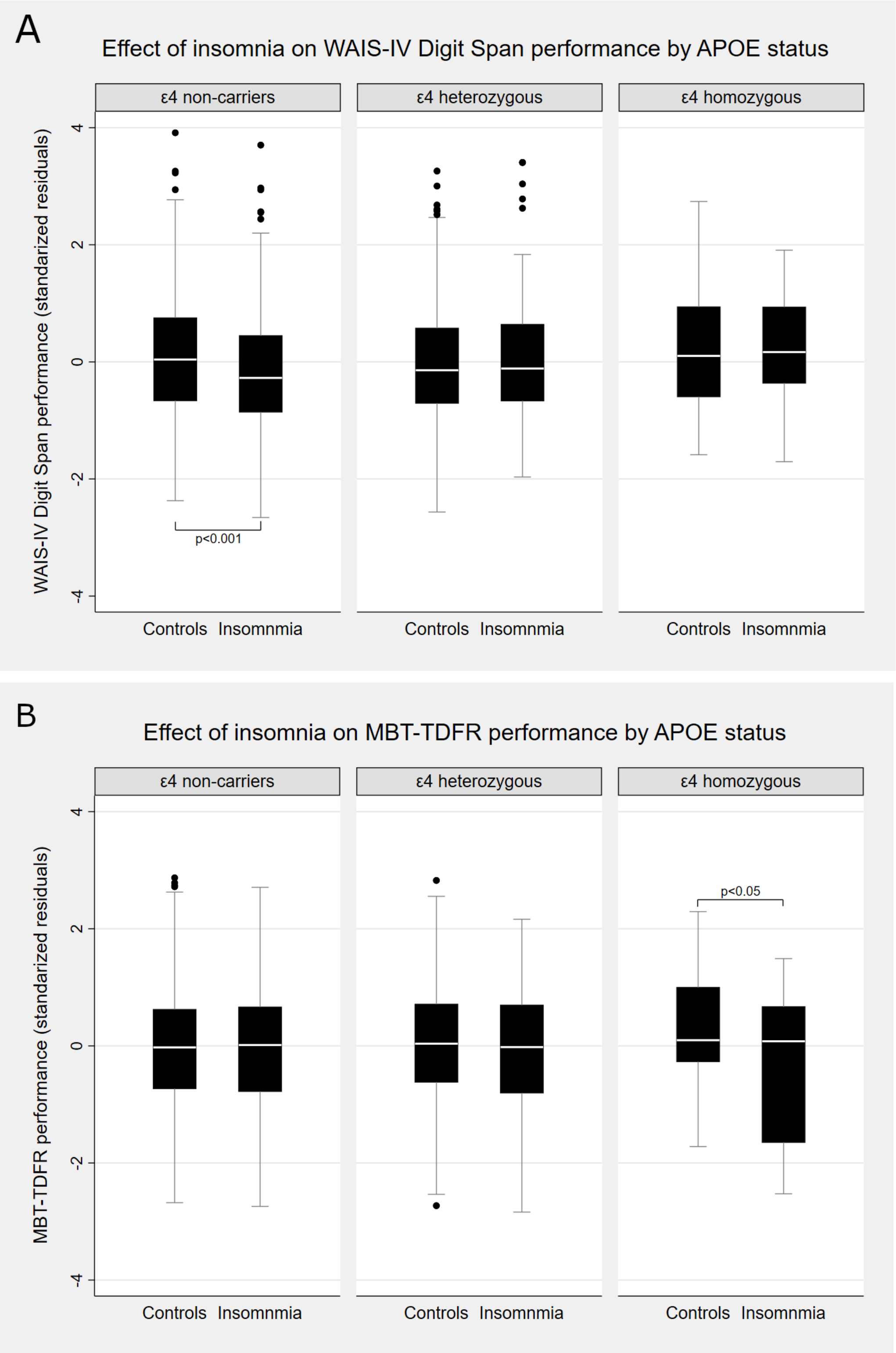

Graphs showing the differential effect of insomnia in cognitive performance depending on the APOE status. Statistically significant negative effects of insomnia are observed in Digit Span performance among APOE-ε4 non-carriers only (A) and in MBT-TDFR performance among APOE-ε4 homozygous only.

Figure S2. Effect of insomnia on gray matter volume (additionally adjusted by hypertension, dyslipidemia and physical activity)

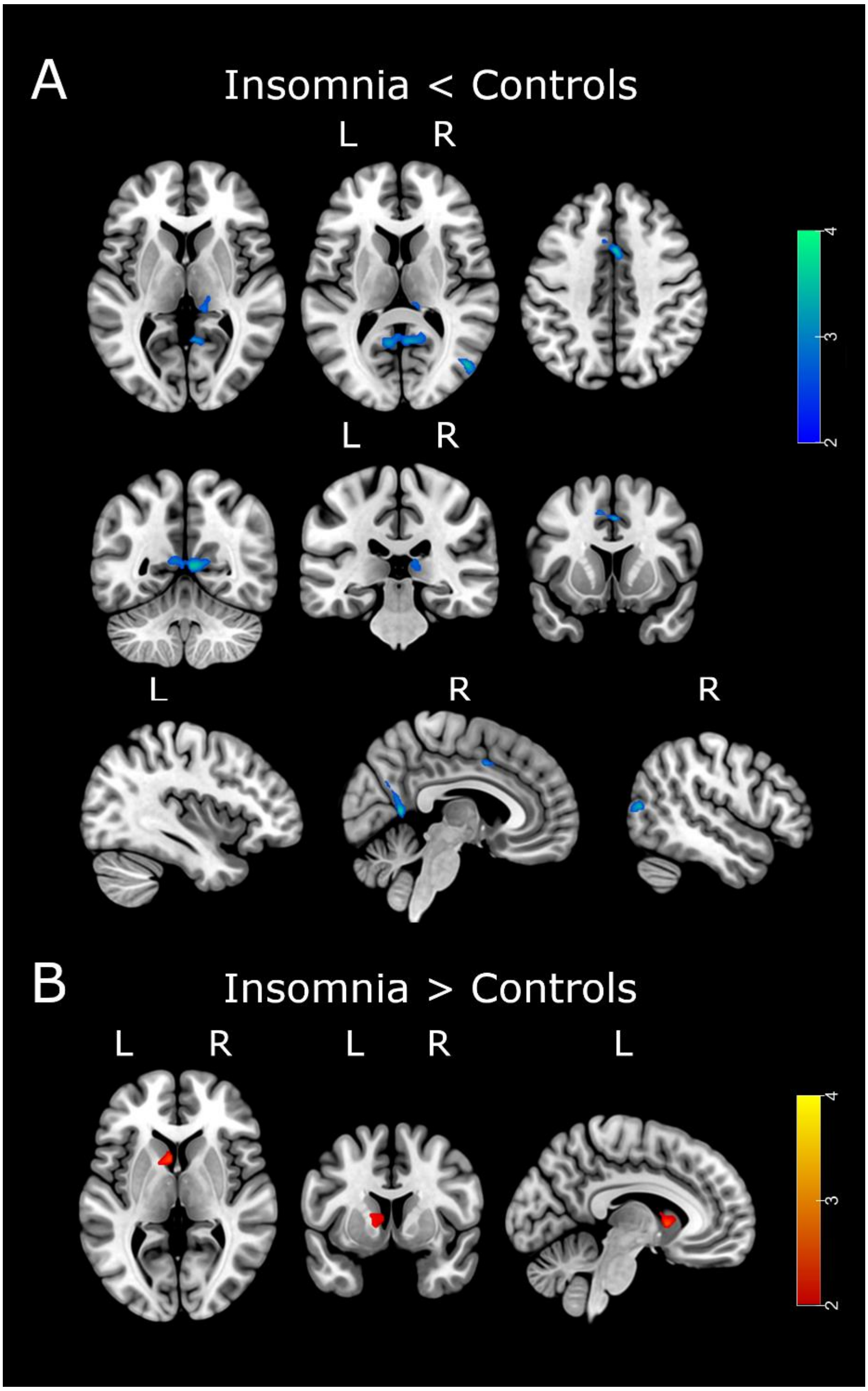

A: Blue-green colored regions show areas with significantly lower volume in participants with insomnia compared with controls (puncorrected<0.005; k=100). B: Red-yellow colored areas show areas with significantly higher brain volume in participants with insomnia compared with controls. L: Left hemisphere. R: right hemisphere.

Figure S3. Effect of insomnia on white matter microstructure (additionally adjusted by hypertension, dyslipidemia and physical activity)

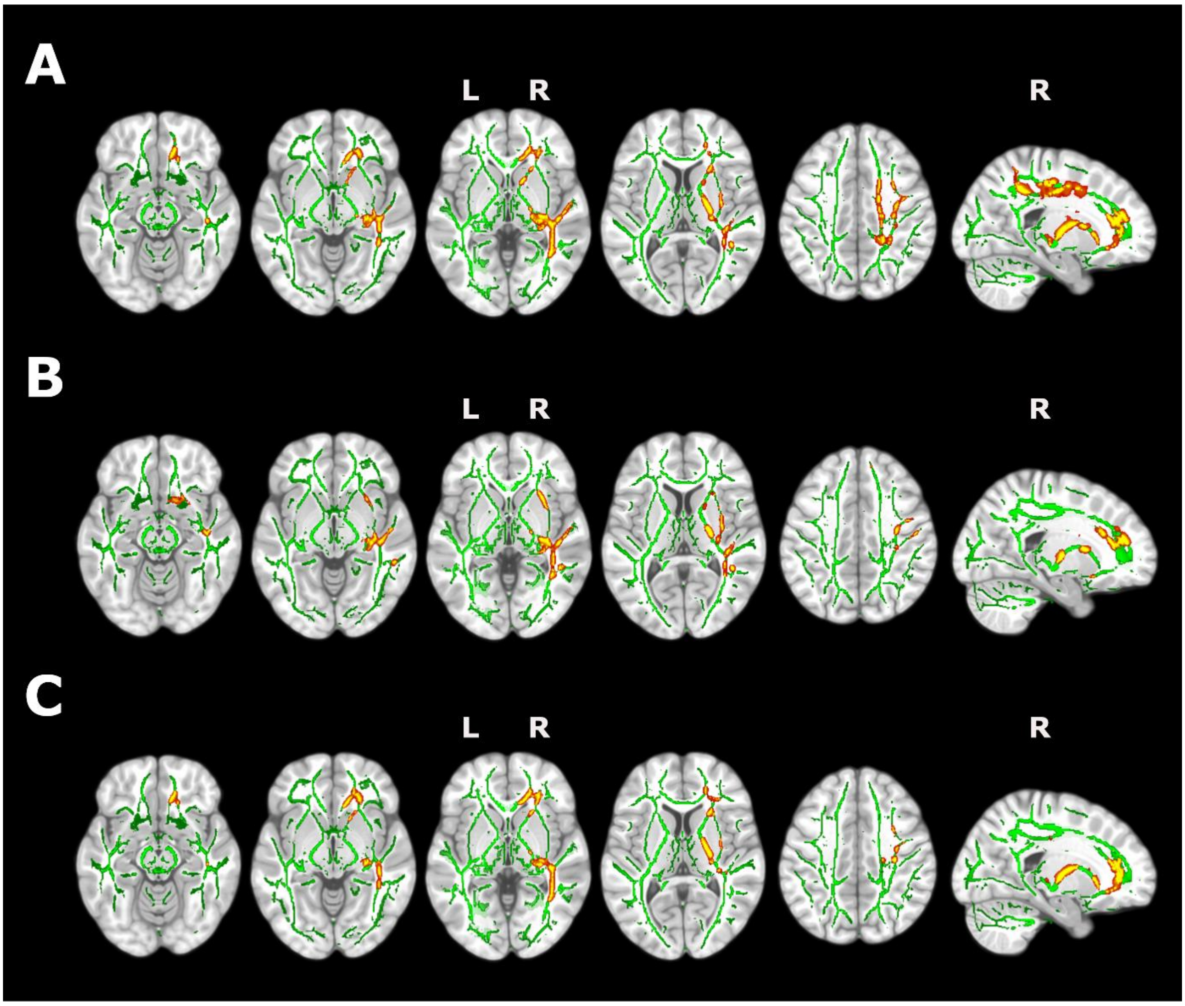

Significant white matter clusters derived from tract-based spatial statistics are represented in red-yellow over the skeletonized white matter tracts (green). Individuals with insomnia showed significantly reduced values of mean (A) and axial (B) diffusivity (FWE corrected p value < 0.05), and a trend for radial diffusivity (C) (FWE corrected p value between 0.05 and 0.1), compared with normal sleepers. L: Left hemisphere. R: right hemisphere.

Table S1. VBM results of the main effect of insomnia, additionally adjusted by hypertension, dyslipidemia and physical activity ($p_{uncorrected}$<0.005; k=100)

Contrast	Anatomical area	Cluster size (k)	Peak-level t-value	$P_{uncorrected}$	MNI coordinates x	y	z
I<C	Bilateral PCC / precuneus	516	3.77	<.001	6	-56	8
	Bilateral middle cingulum	161	3.66	<.001	2	9	42
	Right middle temporal	147	3.59	<.001	48	-72	11
	Right thalamus	112	2.92	.002	11	-30	11
I>C	Left caudate	211	3.03	.001	-6	9	3

I: Insomnia. C: Controls. PCC: posterior cingulate cortex.

Table S2. VBM results of interaction between insomnia and *APOE* genotype ($p_{uncorrected}$<0.005; k=100)

Genetic model	Anatomical area	Cluster size (k)	Peak-level t-value	Puncorrected	MNI coordinates x	y	z
Additive	Left Angular gyrus	656	4.670443058	<.001	-40.5	-63	34.5
	Left Superior Frontal Gyrus	326	4.081087112	<.001	-12	45	34.5
	Right/Left Superior Frontal Gyrus / Middle Cingulate Gyrus	733	3.836615801	<.001	3	21	60
	Left Middle Temporal Gyrus	179	3.824887753	<.001	-61.5	-25.5	-13.5
	Right Hippocampus/Thalamus	207	3.724496841	<.001	13.5	-34.5	7.5
	Left Middle Temporal Gyrus	113	3.549753666	<.001	-54	-51	3
	Left Pre/postcentral Gyrus	259	3.452485323	<.001	-51	-16.5	43.5
	Right Postcentral gyrus	167	3.309949875	<.001	51	-13.5	19.5
	Right Fusiform	166	3.285665512	<.001	27	-66	-7.5
	Right Precentral gyrus	476	3.280437231	<.001	55.5	-12	28.5
	Right Hippocampus	224	3.228650331	<.001	31.5	-34.5	1.5
	Left Thalamus	103	2.902390003	.002	-10.5	-36	4.5
Dominant	Left Middle Temporal Gyrus	321	4.27658606	<.001	-63	-24	-13.5
	Left Postcentral Gyrus	589	4.0510211	<.001	-51	-16.5	45
	Left Inferior Parietal Lobule	634	4.01766729	<.001	-42	-64.5	37.5
	Left Middle Occipital Gyrus	311	3.76290226	<.001	-49.5	-79.5	-4.5
	Right Postcentral Gyrus	489	3.67131305	<.001	58.5	-22.5	30
	Right Postcentral Gyrus	173	3.63615155	<.001	51	-13.5	19.5
	Left Superior/Middle Frontal Gyrus / Middle Cingulate	665	3.6264267	<.001	-6	13.5	57
	Left Middle Temporal Gyrus	123	3.61123657	<.001	-52.5	-49.5	3
	Left Superior Temporal Gyrus	134	3.60978031	<.001	-42	-51	19.5
	Right Thalamus / Right hippocampus	162	3.58622289	<.001	12	-34.5	6
	Left Frontal Superior Medial Gyrus	100	3.35894251	<.001	0	49.5	36
Recessive	Left Angular Gyrus	354	4.62541676	<.001	-40.5	-63	34.5
	Left Frontal Superior Gyrus	208	4.21207428	<.001	-12	45	36
	Right/Left Superior Frontal Gyrus	275	3.92293334	<.001	3	21	60
	Right Hippocampus	307	3.43951154	<.001	33	-27	-9
	Right Fusiform Gyrus	231	3.37325096	<.001	25.5	-67.5	-6
	Right Frontal Superior Medial Gyrus	126	3.08985639	.001	12	27	43.5
	Right Supramarginal Gyrus / Right Postcentral Gyrus	158	3.04352212	.001	63	-19.5	39
